# Maternal Vitamin D Status at Delivery and Allergic Outcomes in Early Adolescence: Prospective Findings from the KLOTHO Birth Cohort

**DOI:** 10.3390/nu18081277

**Published:** 2026-04-17

**Authors:** Spyridon N. Karras, Dimitrios G. Goulis, Nikolaos Angelopoulos, Vikentia Harizopoulou, Maria Kypraiou, Antonios Vlastos, Neoklis Georgopoulos, Georgios Mastorakos, Maria Dalamaga

**Affiliations:** 1 Laboratory of Biological Chemistry, Medical School, Aristotle University, 55535 Thessaloniki, Greece; 2 Unit of Reproductive Endocrinology, 1st Department of Obstetrics and Gynecology, Medical School, Faculty of Health Sciences, Aristotle University of Thessaloniki, 54124 Thessaloniki, Greece; dgg@auth.gr; 3 Endocrinology, Diabetes and Metabolism Clinics, Private Practice, 63200 Kavala, Greece; drangelnick@gmail.com; 4 Department of Midwifery, Faculty of Health and Caring Sciences, University of West Attica, 12243 Athens, Greece; 5 Assisting Nature Centre of Reproduction and Genetics, 57001 Thessaloniki, Greece; mariabioanalysis@yahoo.gr; 6 Medical School, Aristotle University, 55535 Thessaloniki, Greece; antonisvlastos1958@gmail.com; 7 Division of Endocrinology, Department of Internal Medicine, School of Health Sciences, University of Patras, 26504 Patras, Greece; neoklisgeorgo@gmail.com; 8 Second Department of Surgery, Medical School, Aretaieio Athens Hospital, National and Kapodistrian University of Athens, 11528 Athens, Greece; gmastorak@med.uoa.gr; 9 Department of Biological Chemistry, School of Medicine, National and Kapodistrian University of Athens, 10679 Athens, Greece

**Keywords:** vitamin D, 25-hydroxyvitamin D, pregnancy, adolescence, allergy, asthma, rhinitis, eczema, birth cohort

## Abstract

Background: Prenatal vitamin D exposure has been proposed as a potential determinant of immune development and subsequent allergic disease risk in offspring; however, long-term cohort data remain inconsistent. Methods: We analyzed data from the KLOTHO birth cohort, including 98 adolescents with available allergic outcome assessment. A maternal–neonatal sub-cohort of mother–child pairs with available maternal and neonatal serum total 25-hydroxyvitamin D_3_ [25(OH)D] measurements at delivery was used for vitamin D analyses. Allergic outcomes included asthma, allergic rhinitis, and eczema in offspring. Associations were evaluated using descriptive statistics, Spearman correlation analyses, and logistic regression models. Results: Maternal 25(OH)D concentrations were not significantly associated with asthma (ρ = 0.075, *p* = 0.652), allergic rhinitis (ρ = 0.100, *p* = 0.556), or eczema (ρ = 0.131, *p* = 0.426). In crude logistic regression models, vitamin D concentrations were not associated with asthma (odds ratio (OR) per 10 nmol/L: 1.07, 95% confidence interval (CI): 0.78–1.48, *p* = 0.67), allergic rhinitis (OR: 1.05, 95% CI: 0.76–1.45, *p* = 0.77), or eczema (OR: 1.17, 95% CI: 0.86–1.60, *p* = 0.31). Adjusted models including maternal age, pre-pregnancy body mass index (BMI), season of delivery, and ultraviolet exposure yielded similar non-significant findings, although analyses were limited by a reduced complete-case sample size. Conclusions: In this prospective cohort with follow-up into early adolescence, vitamin D status at delivery was not associated with asthma, allergic rhinitis, or eczema in offspring. These findings support a lack of statistically significant association; however, potential non-linear relationships should be interpreted cautiously, given the modest sample size.

## 1. Introduction

Vitamin D has increasingly been recognized as an immunomodulatory hormone with pleiotropic effects that extend beyond calcium homeostasis and bone health. Vitamin D receptors are expressed in several immune cell populations, including dendritic cells, macrophages, and activated T lymphocytes, whereas vitamin D signaling has been shown to influence cytokine production, regulatory T-cell activity, T helper cell polarization and differentiation, and immune tolerance mechanisms [1,2]. These immunomodulatory properties have led to the hypothesis that vitamin D may play an important role in allergic disease pathogenesis and may shape fetal immune programming and influence the risk of allergic disease later in life [3,4,5,6]. In addition to its direct immunomodulatory effects, vitamin D may influence immune development through epigenetic mechanisms, including DNA methylation, histone modification, and regulation of gene expression. These pathways may contribute to the programming of immune responses early in life and potentially affect susceptibility to allergic diseases later in childhood and adolescence.

Experimental and epidemiological evidence suggests that prenatal vitamin D exposure may affect immune programming and subsequent susceptibility to asthma, wheezing disorders, eczema, and allergic rhinitis in childhood [7,8]. Several observational cohort studies have investigated the association between maternal vitamin D concentrations during pregnancy and allergic outcomes in offspring. Some studies have reported that lower maternal vitamin D concentrations are associated with an increased risk of wheezing disorders, asthma, or eczema in early childhood [7,8,9,10,11], whereas others have reported that low maternal vitamin D concentrations during pregnancy are associated with wheezing, eczema, or asthma-like symptoms in infancy and early childhood; others have found no association or even suggested non-linear associations. Other prospective studies and meta-analyses have failed to demonstrate a clear relationship between maternal vitamin D status and the development of allergic diseases in children [12,13,14].

These discrepancies may reflect differences in study populations, timing of vitamin D assessment, baseline prevalence of vitamin D deficiency, and differences in assay methodology. Another unresolved issue is the developmental timing of allergic outcome assessments. Many cohorts have focused on infancy or preschool age, when respiratory symptoms and transient wheezing phenotypes may not reflect stable allergic disease. In contrast, adolescence represents a more mature immunological stage in which allergic rhinitis, eczema, and asthma phenotypes are generally more stable and clinically interpretable.

Accordingly, long-term follow-up may provide a clearer perspective on whether prenatal vitamin D exposure exerts persistent effects on allergic morbidity. Long-term follow-up data examining the relationship between prenatal vitamin D exposure and allergic outcomes in adolescence remain limited. Therefore, understanding whether prenatal vitamin D status has persistent effects on allergic disease risk later in life is of particular interest.

The KLOTHO birth cohort was designed to explore the perinatal determinants of child health. Within this framework, maternal and neonatal vitamin D biomarkers were measured at delivery, and offspring were re-evaluated during adolescence. This analysis aimed to investigate whether maternal and neonatal vitamin D status at delivery is associated with allergic outcomes in early adolescence, including asthma symptoms, allergic rhinitis, and eczema.

## 2. Methods

### 2.1. Study Design and Participants

This study is part of the KLOTHO cohort, a prospective observational birth cohort investigating the role of maternal vitamin D status during pregnancy on long-term health outcomes in the offspring. Pregnant women were recruited from the Maternity Unit of the First Department of Obstetrics and Gynecology, Aristotle University of Thessaloniki, Greece, between January and December 2011 [15]. The inclusion criteria were singleton pregnancies and delivery at term (37–42 weeks of gestation). Women were excluded if they had conditions affecting calcium or vitamin D metabolism, including primary hyperparathyroidism, chronic liver disease, hyperthyroidism, inflammatory bowel disease, nephrotic syndrome, osteomalacia, rheumatoid arthritis, or the use of medications known to influence calcium or vitamin D homeostasis. Neonatal exclusion criteria included small-for-gestational-age birth and the presence of major congenital anomalies.

Of the initial cohort of 191 mother–child pairs, 98 offspring with available follow-up data were included in the present analysis. Maternal and neonatal vitamin D measurements at delivery were available for this subset of participants (n = 98) and were used for vitamin D-related analyses.

The study protocol was approved by the Bioethics Committee of Aristotle University of Thessaloniki, and written informed consent was obtained from all participating mothers, both at baseline and at follow-up (approval no. 1/19-12-2011), and the study was conducted in accordance with the Declaration of Helsinki.

### 2.2. Maternal, Neonatal and Adolescent Assessments

Maternal demographic and clinical variables included age at birth, height, pre-pregnancy weight, weight at term, pre-pregnancy and term body mass index (BMI), gestational age, smoking during pregnancy, alcohol consumption during pregnancy, educational level, previous live births, and dietary calcium and vitamin D intake during the third trimester [16]. Maternal anthropometric measurements were obtained using standardized procedures. In addition, maternal sun exposure and skin pigmentation were assessed using structured questionnaires and standardized skin type classification.

Dietary intake during the last month of pregnancy was assessed using a validated semi-quantitative food frequency questionnaire (FFQ) including approximately 150 food items, designed to capture the characteristics of the Mediterranean diet. Daily intake of macro- and micronutrients, including calcium and vitamin D, was estimated using a food composition database adapted for Greek dietary patterns. Ambient ultraviolet B (UVB) exposure during the 45 days preceding delivery and the season of delivery were recorded as environmental correlates of vitamin D status.

Neonatal measurements included sex, birth weight, and birth length. At follow-up, adolescent offspring underwent anthropometric evaluation using standardized procedures. Age was calculated based on the date of birth and timing of the follow-up assessment. Body height and weight were measured, and BMI was calculated as weight (kg) divided by height squared (m^2^) and is presented as an absolute value for descriptive purposes, without classification according to age- and sex-specific reference standards.

Additional anthropometric measurements included head circumference, waist circumference, low waist circumference, and thigh circumference. All measurements were performed by trained personnel using standardized protocols.

Lifestyle characteristics were assessed using structured questionnaires completed during the follow-up. Sleep duration was recorded as the average number of hours of sleep per day. Physical activity was evaluated using the Physical Activity Questionnaire (PAQ), which provides a composite score reflecting habitual activity levels.

### 2.3. Biochemical Measurements

Maternal venous blood was obtained close to delivery and analyzed using liquid chromatography-tandem mass spectrometry [15]. Umbilical cord blood samples were obtained from the umbilical vein immediately after delivery. Serum samples were stored at −20 °C until analysis. The same analytical platform was used for neonatal cord blood samples. Eight vitamin D metabolites were quantified using liquid chromatography–tandem mass spectrometry (LC-MS/MS): vitamin D_2_, vitamin D_3_, 25(OH)D_2_, 25(OH)D_3_, 1α,25(OH)_2_D_2_, 1α,25(OH)_2_D_3_, 3-epi-25(OH)D_2_, 3-epi-25(OH)D_3_, and total 25 (OH)D, which is the sum of 25(OH)D_2_ and 25(OH)D_3_. Total 25-hydroxyvitamin D [25(OH)D], defined as the sum of 25(OH)D_2_ and 25(OH)D_3_, used as the primary biomarker of vitamin D status in all analyses, as it represents the standard clinical indicator of vitamin D sufficiency.

Maternal and neonatal total 25(OH)D concentrations were selected as the primary exposure variables in the current study. Vitamin D categories were defined as <25 nmol/L, 25–50 nmol/L, and >50 nmol/L [17]. Although multiple vitamin D metabolites were quantified using LC-MS/MS, analyses were performed using total 25(OH)D concentrations only.

Maternal blood samples were collected by antecubital venipuncture approximately 30–60 min before delivery. Additional biochemical parameters measured included calcium, phosphorus, and parathyroid hormone (PTH). Offspring blood samples at adolescence were not obtained.

### 2.4. Allergy Outcomes

Allergy outcomes were assessed using a structured questionnaire based on the International Study of Asthma and Allergies in Childhood (ISAAC) methodology [18,19,20,21]. The questionnaire included standardized items addressing wheezing episodes, exercise-induced wheeze, nocturnal cough, physician-diagnosed asthma, symptoms of allergic rhinitis, seasonal rhinoconjunctivitis, and symptoms of eczema (atopic dermatitis). Asthma outcomes were categorized according to symptom severity and physician diagnosis. Allergic rhinitis outcomes were classified based on the presence of nasal symptoms in the absence of respiratory infection and the coexistence of ocular symptoms. Eczema was defined according to the presence of a chronic pruritic rash with characteristic distribution patterns typical of atopic dermatitis, following established ISAAC definitions [20,21].

### 2.5. Statistical Analysis

No formal a priori sample size calculation was performed, as this study is based on a prospective birth cohort with predefined recruitment. Continuous variables are presented as mean ± standard deviation (SD) or median (interquartile range, IQR), as appropriate, while categorical variables are expressed as absolute counts and percentages. Associations between maternal and neonatal total 25-hydroxyvitamin D_3_ [25(OH)D] concentrations at delivery and allergic outcomes were evaluated using Spearman’s rank correlation coefficients. Logistic regression models were used to examine the association between vitamin D concentrations and binary allergic outcomes (asthma, allergic rhinitis, and eczema). Odds ratios (ORs) and 95% confidence intervals (CIs) were calculated per 10 nmol/L increase in total 25(OH)D.

Multivariable models were adjusted for maternal age, pre-pregnancy BMI, season of delivery, and UVB exposure. Due to missing data for UVB exposure and season of delivery, adjusted analyses were based on complete-case samples and were therefore considered exploratory. Pre-pregnancy BMI was included as a covariate due to its established association with both maternal vitamin D status and offspring health outcomes, and was therefore considered a potential confounder.

In addition, category-based analyses were performed by stratifying maternal vitamin D status into predefined groups (<25, 25–50, and >50 nmol/L) and comparing allergic outcomes across categories. Statistical significance was defined as a two-sided *p*-value < 0.05. All analyses were performed using appropriate statistical software. Analyses were conducted using SPSS version 2021 (IBM Corp., Armonk, NY, USA).

## 3. Results

Maternal and neonatal characteristics are presented in Table 1 and Table 2. The mean maternal age at delivery was 33.95 ± 4.93 years, and the mean pre-pregnancy BMI was 23.4 ± 4.6 kg/m^2^, indicating an overall normal-weight population. The mean BMI at term was 28.4 ± 4.2 kg/m^2^. Most pregnancies occurred during a period of low UVB exposure (87.2%), and the mean UVB exposure during the last 45 days of pregnancy was relatively low (0.23 ± 0.09 Wh/m^2^). Reported vitamin D intake during the third trimester was modest (3.02 ± 1.57 μg/day). The mean birth weight was 3341 ± 383 g, with values within the expected range. The mean age of the offspring at follow-up was 12.2 ± 1.0 years (range, 10.9–13.8 years), corresponding to early adolescence. The mean height and weight were 138.4 ± 11.7 cm and 34.4 ± 10.5 kg, respectively, corresponding to a mean BMI of 17.7 ± 3.7 kg/m^2^.

Lifestyle characteristics indicated that adolescents reported an average sleep duration of 9.12 ± 0.85 h per day and a mean physical activity score (PAQ score) of 2.86 ± 0.64, reflecting moderate levels of habitual physical activity. Additional anthropometric measurements demonstrated a mean head circumference of 51.2 ± 7.0 cm, waist circumference of 63.7 ± 12.6 cm, low waist circumference of 72.0 ± 16.1 cm, and thigh circumference of 41.0 ± 9.5 cm, consistent with expected variability in body composition during this developmental stage.

The prevalence of allergic outcomes was 7.7% for asthma (including physician-diagnosed asthma or current wheezing), 17.9% for allergic rhinitis and 23.1% for eczema. Most eczema cases were classified as mild, and no severe eczema cases were observed. Similarly, asthma cases were limited in number and predominantly non-severe.

**Figure 1:**
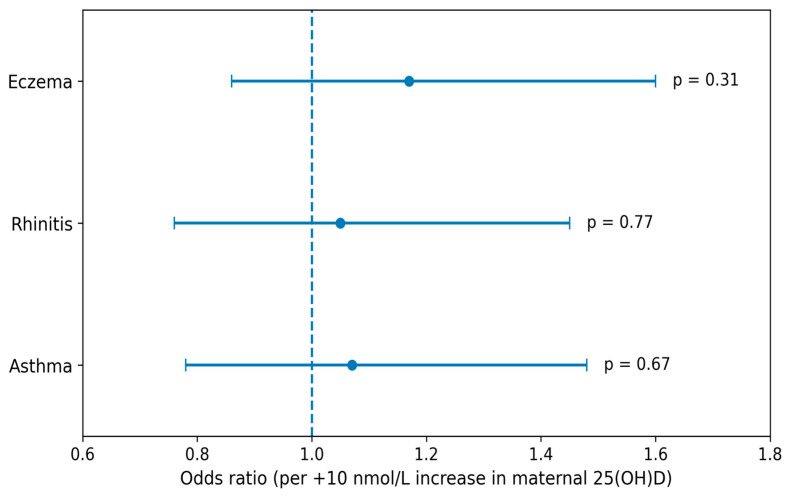
Association between maternal total 25-hydroxyvitamin D [25(OH)D] concentrations at delivery and allergic outcomes in adolescence (n = 98). Odds ratios (ORs) and 95% confidence intervals (CIs) are presented per +10 nmol/L increase in 25(OH)D. The vertical line represents the null value (OR = 1).

**Figure 2:**
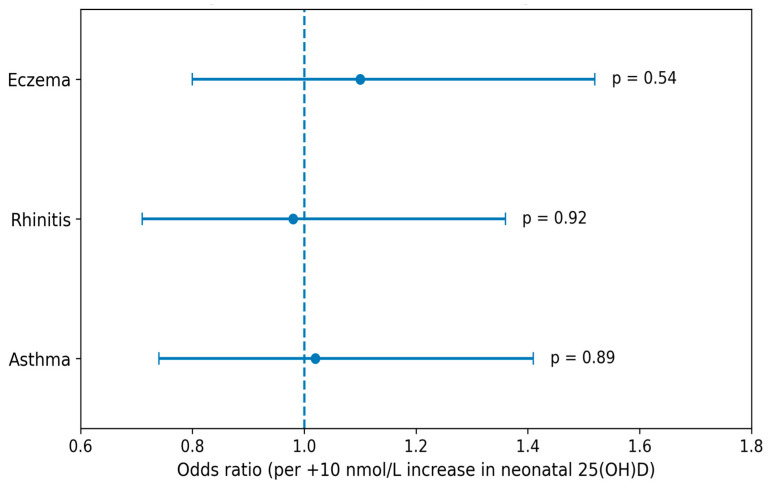
Association between neonatal total 25-hydroxyvitamin D [25(OH)D] concentrations at delivery and allergic outcomes in adolescence (n = 98). Odds ratios (ORs) and 95% confidence intervals (CIs) are presented per +10 nmol/L increase in 25(OH)D. The vertical line represents the null value (OR = 1).

Associations between maternal and neonatal 25(OH)D concentrations at delivery and allergic outcomes are presented in Table 3, Table 4 and Table 5. In correlation analyses (Table 3), neither maternal nor neonatal vitamin D concentrations were significantly associated with asthma, allergic rhinitis, or eczema scores. Correlation coefficients were low and non-significant across all outcomes, indicating weak and inconsistent associations. In crude logistic regression models (Table 4), maternal vitamin D concentrations were not significantly associated with any of the examined outcomes. Specifically, the odds ratio (OR) per 10 nmol/L increase in maternal 25(OH)D was 1.07 (95% CI: 0.78–1.48, *p* = 0.67) for asthma, 1.05 (95% CI: 0.76–1.45, *p* = 0.77) for allergic rhinitis, and 1.17 (95% CI: 0.86–1.60, *p* = 0.31) for eczema. Similar null findings were observed for neonatal vitamin D concentrations. Adjusted models (Table 5), including maternal age, pre-pregnancy BMI, season of delivery, and UVB exposure, yielded comparable results, with no statistically significant associations detected. Some models were unstable due to the limited number of complete cases, particularly for allergic rhinitis and eczema, and should therefore be interpreted with caution. These findings are illustrated in Figure 1 and Figure 2.

## 4. Discussion

In this prospective birth cohort, maternal and neonatal vitamin D status at delivery was not associated with asthma, allergic rhinitis, or eczema in offspring during early adolescence. These findings should be interpreted as evidence of no clear association within this cohort rather than definitive evidence of absence. Larger longitudinal studies with repeated assessments of maternal vitamin D status across pregnancy are required to clarify whether specific gestational windows or non-linear exposure patterns may influence immune programming. The present findings suggest that maternal and neonatal vitamin D status at delivery may not represent major determinants of allergic disease risk during later developmental stages.

Vitamin D has well-established immunomodulatory properties that extend beyond its classical role in calcium homeostasis and bone metabolism [1]. Vitamin D receptors are expressed in multiple immune cell types, including dendritic cells, macrophages, B-lymphocytes, and activated T-lymphocytes. Through these pathways, vitamin D influences antigen presentation, cytokine production, and T-cell differentiation, particularly affecting T helper cell polarization and regulatory T-cell activity [1]. These mechanisms are directly relevant to allergic disease pathogenesis, as vitamin D has been shown to promote immune tolerance and attenuate excessive inflammatory responses. Therefore, prenatal vitamin D exposure has been hypothesized to influence fetal immune development and modify susceptibility to allergic diseases later in life [1,2,3,4].

Early observational studies supported this hypothesis [5,6]. Associations between maternal vitamin D intake or circulating vitamin D concentrations during pregnancy and reduced risk of wheezing disorders or allergic outcomes in offspring have been reported in several cohorts [6,7]. For example, maternal vitamin D exposure has been linked to a lower risk of asthma and allergic rhinitis in childhood [11,12], whereas cord blood vitamin D concentrations have been associated with a reduced risk of wheezing and eczema in early life [22,23,24,25,26,27,28]. These findings initially suggested a potential protective role for prenatal vitamin D in the development of allergic diseases.

However, subsequent epidemiological evidence has been inconsistent. Several large prospective cohort studies have reported no significant associations between maternal vitamin D status and allergic outcomes after adjusting for confounding factors [11,12]. Similarly, studies examining cord blood vitamin D concentrations have yielded mixed results, with some reporting weak or null associations with allergic disease phenotypes [29]. These discrepancies may reflect differences in study design, population characteristics, baseline vitamin D status, and the timing and method of vitamin D assessment. In addition, variability in outcome definitions and follow-up duration further complicates comparisons across studies.

Randomized controlled trials have provided additional insight into this question; however, the findings remain inconclusive [7,9,10]. Prenatal vitamin D supplementation has been associated with a reduction in early-life wheezing in some trials [11,12]; however, its effects on clinically diagnosed asthma and long-term allergic disease outcomes have been less consistent. These findings suggest that while vitamin D may influence early respiratory phenotypes, its impact on persistent allergic diseases may be limited or context-dependent.

The present findings align more closely with studies reporting null or modest associations between maternal vitamin D status and allergic disease outcomes beyond early childhood. One plausible explanation is that allergic disease in adolescence reflects the cumulative effect of multiple genetic and environmental exposures rather than a single prenatal factor. The family history of atopy, microbial exposures in early life, environmental allergens, viral infections, air pollution, and lifestyle-related factors all contribute to the development and persistence of allergic disease. In this context, the contribution of prenatal vitamin D exposure may be relatively small and potentially masked by these other determinants.

Another important consideration is the timing of exposure assessment. In the present study, maternal vitamin D concentrations were measured at delivery, reflecting late gestational status. However, experimental and epidemiological evidence suggests that earlier stages of pregnancy may represent critical windows for immune system programming and lung development [30,31,32,33,34,35]. Vitamin D concentrations can fluctuate substantially during pregnancy due to seasonal variations, sun exposure, and dietary intake. Therefore, a single measurement at delivery may not adequately capture overall maternal vitamin D exposure during pregnancy or during critical developmental periods [32,33,34,35].

A notable strength of this study is its relatively long follow-up period, extending into early adolescence. Many previous studies have focused on infancy or early childhood [5,6,7,8,9], where respiratory symptoms, such as wheezing, are common but often transient and may not reflect established allergic diseases. In contrast, adolescence represents a more stable stage for the assessment of asthma, allergic rhinitis, and eczema phenotypes. The absence of an association at this stage provides important evidence suggesting that prenatal vitamin D status may not have a strong or lasting impact on clinically relevant allergic disease outcomes.

This study also benefited from detailed maternal and neonatal characterization within a well-defined birth cohort. Previous analyses from the KLOTHO cohort have demonstrated strong correlations between maternal and neonatal vitamin D concentrations, supporting the validity of maternal vitamin D status as a proxy for fetal exposure during late gestation. Nevertheless, allergic disease development is a multifactorial process involving complex interactions between genetic susceptibility, immune maturation, and environmental exposures across the life course [36].

Emerging evidence suggests that vitamin D may influence immune development through interactions with the early-life microbiome. Vitamin D has been implicated in the regulation of epithelial barrier function and innate immune responses, which may in turn affect microbial colonization and host–microbiome interactions. Given the established role of the microbiome in immune tolerance and allergic disease susceptibility, this represents a potential pathway linking prenatal vitamin D exposure to long-term health outcomes. However, current evidence is limited, and further research is needed to elucidate these mechanisms.

Despite these strengths, several limitations should be considered. First, the sample size for vitamin D analyses was relatively modest, which may have limited the statistical power to detect small associations. Second, exposure assessment was based on a single maternal vitamin D measurement at delivery, which may not reflect exposure during earlier or more critical stages of pregnancy. Third, allergic outcomes were assessed using standardized ISAAC-based questionnaires rather than clinical re-evaluation, introducing the possibility of misclassification, although these tools are widely validated in epidemiological research.

Another consideration is the potential for non-linear associations between vitamin D status and allergic outcomes. Some studies have reported U-shaped relationships, suggesting that both low and high vitamin D concentrations may be associated with altered immune responses [12]. Although exploratory analyses in the present study suggested variability across vitamin D categories, no consistent or statistically robust pattern was observed. Larger studies are required to rigorously evaluate potential non-linear effects.

In addition, pre-pregnancy BMI may represent both a confounder and a potential intermediate factor in the relationship between maternal vitamin D status and offspring health outcomes. Although it was treated as a confounder in the present analyses, this dual role should be acknowledged when interpreting the findings.

BMI in adolescence was expressed as an absolute value (kg/m^2^) rather than using age- and sex-specific reference standards. Although this approach limits the interpretation of BMI in relation to growth status, BMI was not a primary exposure or outcome variable in this study and was included for descriptive purposes only. The interpretation of BMI in adolescents is limited by the absence of age- and sex-specific classification according to WHO growth references. However, BMI was included for descriptive purposes only and was not used for inferential analyses.

The relatively small sample size and the substantial loss to follow-up represent important limitations of the present study. Attrition may have introduced potential selection bias, and the reduced sample size may have limited the statistical power to detect small or moderate associations. Therefore, the findings should be interpreted with caution. Nevertheless, the prospective design, the availability of both maternal and neonatal vitamin D measurements at delivery, and the long-term follow-up into adolescence constitute important strengths of this analysis. No formal a priori sample size calculation was performed, as this study is based on a prospective birth cohort with predefined recruitment.

Finally, the relatively low prevalence of allergic outcomes, particularly asthma, may have limited the statistical power of the analyses and reduced the ability to detect small or moderate associations. This may also explain the wide confidence intervals observed in some models. Therefore, the findings should be interpreted with caution.

The interpretation of the present null findings should be considered in the context of limited statistical power and the potential for type II error. The relatively small sample size and the low number of outcome events, particularly for asthma, may have reduced the ability to detect small or moderate associations. Therefore, the absence of statistically significant findings should not be interpreted as definitive evidence of no effect. Instead, these results suggest that any potential association between maternal or neonatal vitamin D status and allergic outcomes in adolescence is likely to be modest and may require larger cohorts to be reliably detected.

From a clinical perspective, these findings indicate that vitamin D status at delivery is unlikely to represent a strong independent determinant of allergic disease risk in adolescence. However, subtle effects, non-linear relationships, or interactions with other environmental and genetic factors cannot be excluded and warrant further investigation in larger, well-powered longitudinal studies.

Future research should focus on larger prospective cohorts with repeated vitamin D measurements throughout pregnancy, comprehensive assessment of environmental exposures, and detailed phenotyping of allergic outcomes across different developmental stages. Integrating genetic, immunological, and microbiome-related data may further enhance our understanding of the role of vitamin D in immune programming.

## 5. Conclusions

In conclusion, this prospective analysis of the KLOTHO birth cohort did not demonstrate a significant association between maternal and neonatal total 25(OH)D concentrations at delivery and allergic outcomes in early adolescence. These findings support a predominantly null association and highlight the need for further research to explore potential subtle or context-specific effects.

## Figures and Tables

**Table 1:** Demographic and clinical characteristics of mothers at delivery. Data are presented as mean ± standard deviation (SD), median (interquartile range, IQR), or n (%), as appropriate. Abbreviations: BMI, body mass index; PTH, parathyroid hormone; 25(OH)D, 25-hydroxyvitamin D.

Variable	(n = 98)
Age at birth (years)	34.0 ± 4.9
Height (cm)	165.6 ± 6.8
Weight pre-pregnancy (kg)	64.3 ± 12.9
Weight at term (kg)	77.8 ± 12.7
BMI pre-pregnancy (kg/m^2^)	23.4 ± 4.6
BMI at term (kg/m^2^)	28.4 ± 4.2
Weeks of gestation	38.2 ± 1.4
Smoking during pregnancy	4 (10.3%)
Alcohol during pregnancy	6 (15.4%)
Previous live births	0.0 (0.0–1.0)
Ca intake trimester 3 (mg/d)	880 ± 474
Vitamin D intake trimester 3 (μg/d)	3.02 ± 1.57
UVB exposure (Wh/m^2^)-45 d	0.23 ± 0.09
Season: low UVB period	34 (87.2%)
Maternal 25(OH)D (nmol/L)	47.2 ± 26.1
Calcium (mg/dL)	9.2 ± 1.2
PTH (pg/mL)	31.9 ± 13.0

**Table 2:** Anthropometric and clinical characteristics of offspring at birth and during adolescence. Data are presented as mean ± standard deviation (SD), median (interquartile range, IQR), or n (%), as appropriate. Abbreviations: BMI, body mass index; PTH, parathyroid hormone; 25(OH)D, 25-hydroxyvitamin D.

Variable	(n = 98)
Sex: female/male	47/51
Age (y)	12.2 ± 1.0
Birth weight (g)	3341 ± 383
Birth length (cm)	50.9 ± 2.0
Height at adolescence (cm)	138.4 ± 11.7
Weight at adolescence (kg)	34.4 ± 10.5
BMI at adolescence (kg/m^2^)	17.7 ± 3.7
Sleep (h/day)	9.12 ± 0.85
Physical activity (PAQ score)	2.86 ± 0.64
Head circumference (cm)	51.2 ± 7.0
Waist circumference (cm)	63.7 ± 12.6
Low waist circumference (cm)	72.0 ± 16.1
Thigh circumference (cm)	41.0 ± 9.5
Neonatal 25(OH)D (nmol/L)	35.0 ± 20.0
Calcium at birth (mg/dL)	8.1 ± 0.8
PTH at birth (pg/mL)	11.9 ± 3.0

**Table 3:** Spearman correlation coefficients (ρ) between maternal and neonatal total 25-hydroxyvitamin D [25(OH)D] concentrations at delivery and allergic outcome scores in adolescence. Abbreviations: ρ, Spearman correlation coefficient.

Outcome	n	Maternal ρ	*p*	Neonatal ρ	*p*
Asthma score	98	0.075	0.652	0.060	0.710
Rhinitis score	97	0.100	0.556	0.082	0.620
Eczema score	98	0.131	0.426	0.110	0.500

**Table 4:** Crude logistic regression models examining the association between maternal and neonatal total 25-hydroxyvitamin D [25(OH)D] concentrations at delivery and allergic outcomes in adolescence. Odds ratios (ORs) and 95% confidence intervals (CIs) are presented per +10 nmol/L increase in 25(OH)D. Abbreviations: OR, odds ratio; CI, confidence interval.

Outcome	n	Maternal OR	*p*	Neonatal OR	*p*
Any asthma	98	1.07 (0.78–1.48)	0.670	1.02 (0.74–1.41)	0.890
Any rhinitis	97	1.05 (0.76–1.45)	0.770	0.98 (0.71–1.36)	0.920
Any eczema	98	1.17 (0.86–1.60)	0.310	1.10 (0.80–1.52)	0.540

**Table 5:** Multivariable logistic regression models examining the association between maternal and neonatal total 25-hydroxyvitamin D [25(OH)D] concentrations at delivery and allergic outcomes in adolescence, adjusted for maternal age, pre-pregnancy BMI, season of delivery, and ultraviolet B (UVB) exposure. Odds ratios (ORs) and 95% confidence intervals (CIs) are presented per +10 nmol/L increase in 25(OH)D. Abbreviations: OR, odds ratio; CI, confidence interval.

Outcome	n	Maternal OR	*p*	Neonatal OR	*p*
Any asthma	98	1.27 (0.56–2.87)	0.560	1.18 (0.52–2.66)	0.690
Any rhinitis	97	Not estimable	-	Not estimable	-
Any eczema	98	Not estimable	-	Not estimable	-

## Data Availability

The datasets presented in this article are not readily available because the data are part of an ongoing study.
